# The integrated nurse self-leadership and caring scale: development and psychometric evaluation

**DOI:** 10.1016/j.ijnsa.2026.100556

**Published:** 2026-05-11

**Authors:** Mahmud Ady Yuwanto, Iyus Yosep, Iqbal Pramukti, Ati Surya Mediawati

**Affiliations:** a Doctoral Student in Nursing, Faculty of Medicine, Universitas Padjadjaran, Bandung, Indonesia; b Department of Mental Health Nursing, Faculty of Nursing, Universitas Padjadjaran, Bandung, Indonesia; c Departement of Community Health Nursing, Faculty of Nursing, Universitas Padjadjaran, Bandung, Indonesia; d Departement of Fundamental Nursing, Faculty of Nursing, Universitas Padjadjaran, Bandung, Indonesia; e Departement of Fundamental Nursing, Faculty of Health Sciences, Universitas dr. Soebandi, Jember, Indonesia

**Keywords:** Nursing leadership, Self-leadership, Caring, Psychometrics, Instrument development

## Abstract

**Background:**

**:** Nurses require both self-leadership and caring capacities to deliver safe, compassionate, and adaptive care. Self-leadership refers to an individual’s ability to regulate thoughts, behaviors, and motivation to achieve professional goals. Existing instruments typically measure these constructs separately, limiting holistic assessment.

**Objective:**

**:** We aimed to develop and psychometrically evaluate the Integrated Nurse Self-Leadership and Caring Scale.

**Methods:**

**:** A methodological study was conducted in two phases: instrument development and psychometric testing. A 30-item pool was generated from self-leadership and caring theories and refined through expert review and cognitive debriefing. Data were collected from 169 Indonesian nurses and final-year professional nursing students. Internal consistency was assessed with Cronbach’s α, while construct validity was examined using exploratory factor analysis (EFA).

**Results:**

**:** Respondents had a mean age of 34.6 years (standard deviation = 8.9), with 72.8% employed as professional nurses. Cronbach’s α values ranged from 0.68 to 0.87 across six domains, and the overall scale demonstrated excellent reliability (α = 0.92). The Kaiser–Meyer–Olkin value was 0.887, and Bartlett’s test was significant (*p* < .001). EFA supported a six-factor structure, accounting for 64.0% of total variance, with 29 of 30 items meeting the loading threshold.

**Conclusion:**

**:** We found that the Integrated Nurse Self-Leadership and Caring Scale demonstrated promising reliability and construct validity among Indonesian nurses, providing preliminary evidence of its usefulness for assessing the integration of self-leadership and caring within this context. Future researchers should extend validation through confirmatory and predictive analyses.

**What is already known about the topic**

• Instruments measuring self-leadership and caring in nursing are available but assess these constructs separately.
• Self-leadership supports nurses’ self-regulation and motivation, while caring underpins relational and ethical practice.
• No integrated measure exists to capture the combined attributes required in contemporary nursing roles.

Alt-text: Unlabelled box dummy alt text

**What this paper adds**

• We developed the Integrated Nurse Self-Leadership and Caring Scale as the first tool to measure self-leadership and caring as a unified construct.
• We provided psychometric evidence supporting a six-domain structure with strong reliability and construct validity.
• We have made the Integrated Nurse Self-Leadership and Caring Scale publicly accessible in bilingual format to support replication, cross-cultural validation, and application in education and practice.

Alt-text: Unlabelled box dummy alt text

## 1 Introduction

Nursing practice in the 21st century is increasingly challenged by complex healthcare environments, rapid technological advancements, and rising patient expectations (Jenkins, 2022; Roy, 2021). Nurses are required not only to provide technical and evidence-based care but also to demonstrate strong personal and professional qualities that ensure safety, quality, and compassion in patient outcomes (Hou et al., 2024; Li, 2025; Wells-Beede et al., 2023). These dual expectations highlight the critical role of self-leadership in an individual’s ability to regulate thoughts, behaviors, and motivation to achieve personal and professional goals and caring, the ethical and relational foundation of nursing practice (Jana et al., 2023; Pursio et al., 2025).

Globally, issues such as nurse burnout, turnover, and declining engagement have reached high levels, with implications for patient safety and organizational sustainability (Pursio et al., 2023). The World Health Organization has emphasized the importance of strengthening the nursing workforce to meet global health demands, particularly in light of increasing chronic disease burdens and aging populations (Labrague et al., 2021). Self-leadership equips nurses to remain resilient, adaptive, and motivated under pressure, while caring ensures that the humanistic essence of nursing is preserved despite systemic challenges (Bejster et al., 2020; Jameson et al., 2022). Without intentional development and measurement of these attributes, healthcare systems risk compromising both workforce well-being and patient-centered care (Notarnicola et al., 2024; Wynn et al., 2023).

Historically, leadership in nursing has been conceptualized through hierarchical or managerial perspectives, often emphasizing external control and organizational authority (Labrague et al., 2021; Pursio et al., 2025). However, contemporary nursing recognizes that self-leadership must also occur at the individual level, where self-regulation, self-motivation, and reflective practice empower nurses to take initiative and sustain performance (Bracht et al., 2021; Manz, 1986). Parallel to this, caring science has developed as a central paradigm, stressing the relational, spiritual, and moral responsibilities of nurses toward patients and families (Alsadaan et al., 2023; Zhao et al., 2025). Both constructs have been extensively studied but usually in isolation from one another.

A review of existing instruments reveals that validated scales on self-leadership (e.g., Revised Self-Leadership Questionnaire) have been applied primarily in business or general organizational contexts, with limited adaptation to nursing (Manz, 1986; Neck and Houghton, 2006). Similarly, tools measuring caring (e.g., Caring Behaviors Inventory, Caring Dimensions Inventory) focus largely on relational aspects without considering the intrapersonal processes that sustain them (Swanson, 1991; Watson, 2012). This separation creates a conceptual gap; while self-leadership enables nurses to manage themselves effectively, caring translates these capacities into meaningful, patient-centered interactions (Chen et al., 2024; Šabanė et al., 2022). In practice, these constructs are inseparable; self-leadership without caring risks becoming task-focused, while caring without self-leadership may lack sustainability in high-pressure environments.

The research gap, therefore, lies in the absence of an integrated measurement tool that captures self-leadership and caring as a unified construct (Šabanė et al., 2022). Specifically, no validated instrument currently exists to assess the integration of self-leadership and caring among clinical nurses within the Indonesian healthcare context.

Indonesia represents one of the largest nursing workforces in Southeast Asia, with diverse educational pathways and clinical practice settings. The rapid expansion of healthcare services, combined with workforce distribution challenges and increasing patient complexity, underscores the need for culturally relevant tools to assess professional competencies. Developing an instrument within the Indonesian context ensures conceptual, linguistic, and contextual appropriateness for local clinical nurses.

Without such a tool, it remains difficult to comprehensively evaluate how nurses simultaneously regulate themselves and engage in authentic caring, limiting both educational assessment and managerial efforts to strengthen leadership and caring climates in clinical practice (Notarnicola et al., 2024; Ozdemir et al., 2025).

The novelty of the present study lies in the development of the Integrated Nurse Self-Leadership and Caring Scale. Unlike existing leadership instruments that focus primarily on managerial or integral leadership constructs, we have reported on the development of a new instrument rather than the adaptation or further psychometric testing of an existing scale. The Integrated Nurse Self-Leadership and Caring Scale was conceptualized to encompass six domains that bridge intrapersonal and interpersonal dimensions of nursing practice (Manz, 1986; Neck and Houghton, 2006; Swanson, 1991; Watson, 2012). These domains were derived through a theoretical synthesis of self-leadership and caring frameworks, aiming to capture both self-regulatory processes and relational caring behaviors essential to contemporary nursing.

By enabling systematic assessment of both self-regulatory and caring capacities, the instrument may support professional development initiatives aimed at strengthening patient-centered care, nurse resilience, and ethical practice within Indonesian healthcare institutions. Therefore, the aim of this study was to develop and psychometrically evaluate the Integrated Nurse Self-Leadership and Caring Scale among Indonesian clinical nurses. Specifically, we sought to examine the internal consistency reliability and exploratory factor structure of the proposed six-domain instrument, thereby contributing a theoretically grounded and empirically tested tool to support nursing education, practice, and research.

## 2 Methods

### 2.1 Study design

We employed a methodological, cross-sectional design focusing on instrument development and psychometric testing. The scale development process followed established methodological guidelines for instrument construction and validation, particularly those outlined by DeVellis (2017), which emphasize systematic item generation, content validation, pilot testing, and exploratory psychometric evaluation.

#### 2.1.1 Phase I: instrument development

##### 2.1.1.1 Item generation and theoretical foundation

The Integrated Nurse Self-Leadership and Caring Scale was developed through a theoretical synthesis of self-leadership theory (Manz, 1986; Neck and Houghton, 2006) and caring theory (Swanson, 1991; Watson, 2012). Based on this synthesis, six conceptual domains were identified to represent integrated self-leadership and caring capacities in nursing practice.

An initial pool of 30 items (five items per domain) was generated to reflect both intrapersonal self-regulatory processes and interpersonal caring behaviors. All items were rated using a 5-point Likert scale (1= strongly disagree to 5= strongly agree), selected to balance sensitivity, respondent burden, and consistency with existing nursing leadership and caring instruments.

##### 2.1.1.2 Content validity

Content validation was conducted by a panel of five experts with doctoral qualifications and at least 10 years of experience in nursing leadership, caring science, or psychometric research. Experts independently evaluated item relevance using a 4-point scale (1= not relevant to 4= highly relevant). Specifically, the item-level content validity index (I-CVI) was computed as the proportion of experts assigning a rating of either 3 (quite relevant) or 4 (highly relevant) to each item, thereby reflecting the degree of expert agreement regarding item relevance. The scale-level content validity index (S-CVI/Ave) was derived by calculating the mean of all item-level content validity index (I-CVI) values across items, representing the overall proportion of items rated as content-valid by the expert panel. The scale-level content validity index (S-CVI/Ave) was 0.92, indicating excellent overall content validity of the instrument.

A single round of expert review was conducted, as recommended for early-stage instrument development. Items with I-CVI values below the acceptable threshold were revised for clarity based on expert feedback.

##### 2.1.1.3 Cognitive debriefing and pilot testing

Cognitive debriefing was conducted with 10 practicing nurses to assess clarity, readability, and cultural appropriateness of the items. Minor wording revisions were made accordingly. Subsequently, a pilot test was carried out with 30 nurses working in hospital settings to evaluate item comprehension, response patterns, and completion time. No items demonstrated floor or ceiling effects, and all items were retained for the main study.

#### 2.1.2 Phase II: psychometric testing

##### 2.1.2.1 Participants and sampling

A convenience sample of 169 participants was recruited from clinical and academic nursing settings in Indonesia, including three hospitals and two academic nursing institutions, to ensure variability in clinical and educational contexts. The sample consisted of 123 employed clinical nurses, defined as registered nurses holding at least a diploma or bachelor-level nursing qualification and actively engaged in direct patient care within hospital settings, and 46 final-year professional nursing students enrolled in a structured professional nursing program that prepares graduates for clinical registration. Inclusion criteria were: (1) age ≥ 18 years and (2) active engagement in clinical nursing practice or professional nursing education.

Convenience sampling was chosen due to feasibility considerations and the exploratory nature of the psychometric evaluation. The sample size met commonly-accepted recommendations for exploratory factor analysis (EFA), exceeding a 5:1 respondent-to-item ratio (DeVellis, 2017). Although a priori power analysis was not conducted, the achieved sample size was deemed adequate for initial construct exploration.

##### 2.1.2.2 Data collection

Data were collected over a 4-week period in October 2025 using a secure online survey platform. Participants received study information electronically and provided informed consent prior to participation. The response rate for the main survey was 82% based on distributed invitations.

### 2.2 Data analysis

Descriptive statistics were used to summarise participant characteristics and item-level responses, including frequencies, percentages, means, and standard deviations. Internal consistency reliability was assessed using Cronbach’s alpha coefficients for each subscale and for the total scale.

The sample size met commonly-accepted recommendations for EFA. Factor extraction was conducted using principal component analysis with varimax rotation, following established psychometric guidelines (DeVellis, 2017; Kyriazos and Poga, 2023). Sampling adequacy was evaluated using the Kaiser–Meyer–Olkin (KMO) measure and Bartlett’s test of sphericity. Items with factor loadings ≥ 0.40 were retained as acceptable indicators of their respective domains. All analyses were conducted using IBM SPSS Statistics, version 26.0.

### 2.3 Ethical considerations

Ethical approval for this study was obtained from the Research Ethics Committee of Universitas Padjadjaran (Approval No. 902/UN6.KEP/EC/2025). All study procedures complied with the ethical standards of the institutional review board and adhered to the principles of the Declaration of Helsinki. Prior to participation, participants received comprehensive information regarding the study objectives, procedures, and the voluntary nature of their involvement. Electronic written informed consent was obtained from all participants. Participant confidentiality and anonymity were rigorously safeguarded throughout data collection and analysis, with all data securely stored and used exclusively for research purposes.

## 3 Results

All items of the Integrated Nurse Self-Leadership and Caring Scale were rated on a 5-point Likert scale, ranging from 1 (strongly disagree) to 5 (strongly agree), with higher scores indicating stronger integration of self-leadership and caring attributes.

### 3.1 Participant characteristics

A total of 169 participants were included in the study. Most participants were female (*n* = 138, 81.7%). This distribution reflects the demographic composition of the nursing workforce in Indonesia, where female nurses constitute the predominant proportion of practicing professionals.

In terms of professional status, about three quarters of participants were employed clinical nurses, while approximately one quarter were final-year professional nursing students. Participants represented a broad range of clinical experience, with many reporting >10 years of practice and others having <1 year of experience (Table 1).

**Table 1:** Participant characteristics (*N* = 169).

Variable	*n* (%)
**Sex**	
Female	138 (81.7)
Male	31 (18.3)
**Profesional status**	
Clinical nurses	123 (72.8)
Final-year professional nursing students	46 (27.2)
**Position**	
Employed nurse	123 (72.8)
Final-year students of the professional nursing program	46 (27.2)
**Clinical experience**	
<1 year	47 (27.8)
1–5 years	21 (12.4)
6–10 years	15 (8.9)
>10 years	86 (50.9)
**Age (years)**	Mean= 34.6 (SD= 8.9)

Note: *N*= total sample size; *n*= number of participants; %= percentage; SD= standard deviation.

### 3.2 Descriptive statistics and internal consistency

The Integrated Nurse Self-Leadership and Caring Scale demonstrated strong overall internal consistency, supporting its reliability as a multidimensional measure of self-leadership and caring capacities. Across dimensions, the findings indicate consistently positive responses, suggesting that the construct is coherently represented through both intrapersonal self-leadership processes and relational caring components. Dimensions reflecting empathic presence, supportive care environments, and values-oriented caring were particularly salient, underscoring the centrality of relational and ethical elements within the integrated model.

At the scale level, the instrument functioned as a unified measure, with acceptable to high reliability across domains. Together, these findings support the internal coherence of the scale and provide preliminary evidence that the integrated self-leadership and caring framework can be meaningfully operationalized for use in nursing research and practice in Indonesia (Table 2).

**Table 2:** Descriptive statistics and Cronbach’s alpha of integrated nurse self-leadership and caring scale.

Dimension	Items	Mean	SD	Cronbach’s α
Self-regulation & goal orientation	5	4.29	0.65	0.75
Positive cognition & motivation	5	4.37	0.60	0.70
Reflective practice & adaptability	5	4.38	0.54	0.68
Empathic presence & trusting care	5	4.67	0.41	0.87
Hope, spirituality & empowerment	5	4.56	0.50	0.85
Supportive actions & care environment	5	4.64	0.42	0.84
**Total scale**	30	4.47	0.42	0.92

Note: SD= standard deviation; α= Cronbach’s alpha.

### 3.3 Construct validity

The factor analysis revealed a clear and interpretable multidimensional structure underlying the Integrated Nurse Self-Leadership and Caring Scale. The extracted factors collectively accounted for a substantial proportion of the overall variance, indicating that the construct was well-represented across multiple dimensions. The dominant contribution of the initial factor suggested the presence of a strong overarching dimension, while subsequent factors contributed incrementally, reflecting meaningful subcomponents of self-leadership and caring within the integrated framework.

Overall, the pattern of variance distribution supported the conceptual coherence of the scale and aligned with its theoretical foundation, which integrates intrapersonal self-leadership processes with relational and values-based caring elements. These findings provide further evidence that the scale captured both a unifying construct and distinct, theoretically grounded dimensions relevant to nursing practice and research (Table 3).

**Table 3:** Eigenvalues and variance explained of integrated nurse self-leadership and caring scale (Top 6 factors).

Factor	Eigenvalue	% Variance	Cumulative %
1	11.22	37.4	37.4
2	2.63	8.8	46.1
3	1.50	5.0	51.1
4	1.48	4.9	56.1
5	1.30	4.3	60.4
6	1.10	3.6	64.0

### 3.4 Factor loadings

Varimax rotation yielded an interpretable factor solution. Twenty-nine items demonstrated satisfactory primary loadings (≥0.40). One item, “*I use creativity in solving patient care problems*” from the Supportive Actions and Care Environment domain, had a borderline loading of 0.37. This item may require revision in future studies (Table 4).

**Table 4:** Primary factor loadings of integrated nurse self-leadership and caring scale items (*N* = 169).

Dimension	Item	Primary Loading
**Self-Regulation and Goal Orientation**	I set daily work targets to guide my nursing practice.	0.72
	I monitor my progress in achieving clinical goals.	0.68
	I adjust my actions to remain focused on priorities.	0.65
	I remind myself to stay disciplined in clinical tasks.	0.61
	I plan strategies to accomplish nursing responsibilities.	0.59
**Positive Cognition and Motivation**	I speak positively to myself in stressful situations.	0.71
	I encourage myself to remain motivated during challenges.	0.69
	I replace negative thoughts with constructive ones.	0.66
	I build confidence by focusing on my strengths.	0.63
	I visualize success before performing nursing duties.	0.57
**Reflective Practice and Adaptability**	I reflect on my performance after providing care.	0.70
	I learn from mistakes to improve my practice.	0.68
	I adapt quickly to changes in patient conditions.	0.62
	I evaluate the effectiveness of my interventions.	0.58
	I integrate new knowledge into my clinical practice.	0.56
**Empathic Presence and Trusting Care**	I listen actively to patients without judgment.	0.82
	I build trusting relationships with patients.	0.82
	I provide a sense of safety to patients through empathy.	0.79
	I respect each patient’s unique experiences.	0.68
	I am fully present when patients need emotional support.	0.60
**Hope, Spirituality, and Empowerment**	I instill hope in patients during difficult times.	0.76
	I support patients’ spiritual needs when relevant.	0.73
	I help patients find meaning in their experiences.	0.72
	I encourage patients to believe in their recovery.	0.70
	I empower patients to participate actively in their care.	0.68
**Supportive Actions and Care Environment**	I create a comfortable and safe care environment.	0.74
	I collaborate with colleagues to optimize patient care.	0.70
	I provide emotional support to patients and families.	0.69
	I ensure continuity of care across shifts.	0.64
	I use creativity in solving patient care problems.	0.37

Note: Factor loadings ≥ 0.40 were considered acceptable.

## 4 Discussion

In the present study, we developed and psychometrically evaluated the Integrated Nurse Self-Leadership and Caring Scale, a novel instrument designed to capture two fundamental dimensions of nursing practice: self-leadership and caring. We provided empirical evidence supporting the internal consistency and construct validity of the instrument, indicating its potential usefulness in nursing research and practice.

### 4.1 Integration of self-leadership and caring

The six-factor solution confirmed the conceptual framework underpinning the Integrated Nurse Self-Leadership and Caring Scale. Three domains reflected self-leadership (self-regulation and goal orientation, positive cognition and motivation, reflective practice and adaptability), while the remaining three domains addressed caring (empathic presence and trusting care, hope and spirituality, supportive actions and care environment). This alignment between empirical results and theoretical expectations supported the construct validity of the instrument. The integration of these domains may be important in contemporary nursing, where professionals must simultaneously regulate themselves and provide holistic, compassionate care (Manz, 1986; Neck and Houghton, 2006; Swanson, 1991; Watson, 2012).

This integrated structure reflects contemporary nursing practice, in which intrapersonal self-regulatory capacities and interpersonal caring behaviors are enacted simultaneously, rather than as isolated competencies. Similar to recent discussions on integrated leadership and caring in nursing, the Integrated Nurse Self-Leadership and Caring Scale conceptualizes leadership not solely as a managerial function but as a self-directed process that sustains caring engagement in complex clinical environments (Zhao et al., 2025).

### 4.2 Reliability of the instrument

Internal consistency analyses demonstrated that the Integrated Nurse Self-Leadership and Caring Scale had promising overall reliability (α= 0.92), comparable to that reported in other nursing leadership and caring instruments. Subscale reliabilities ranged from acceptable to excellent. Two self-leadership domains (positive cognition and reflective practice) yielded Cronbach’s α values slightly below the conventional threshold of 0.70; however, such values are considered acceptable in early-stage instrument development (DeVellis, 2017). Taken together, these findings suggest that the Integrated Nurse Self-Leadership and Caring Scale demonstrates *promising*, rather than definitive, psychometric performance, warranting further validation in larger and more diverse samples.

### 4.3 Construct validity and factor structure

The KMO value of 0.887 and a statistically significant Bartlett’s test of sphericity indicated that the data were appropriate for factor analytic procedures. Exploratory factor analysis yielded a six-factor solution explaining 64.0% of the total variance, a level of explained variance comparable to previous psychometric studies in nursing leadership and caring research (Houghton and Neck, 2002). Consistent with recent methodological studies examining leadership-related constructs in nursing populations, the observed factor structure supports the multidimensional nature of self-leadership and caring as interrelated yet distinct domains (Pursio et al., 2023, 2025).

### 4.4 Item-level considerations

At the item level, 29 of 30 items loaded satisfactorily (≥0.40). For one item, “*I use creativity in solving patient care problems*”, a borderline loading (0.37) was observed. Although this item aligns conceptually with the supportive actions domain, its empirical contribution was weaker. Possible explanations include the abstract wording of the statement or cultural differences in interpreting “creativity” in patient care. While the overall subscale reliability remained strong, this item warrants revision and further testing. Refining its wording to emphasize observable behaviors (e.g., “*I develop innovative approaches to resolve patient care challenges*”) may enhance its discriminative power. Future research should re-evaluate this item using confirmatory factor analysis (CFA).

The Integrated Nurse Self-Leadership and Caring Scale demonstrated promising reliability and validity as a holistic measure of self-leadership and caring integration among Indonesian nurses. To support future research and cross-cultural adaptation, the full version of the Integrated Nurse Self-Leadership and Caring Scale (original Bahasa Indonesia version and English translation) is provided in the **Supplementary Material**.

## 5 Implications for nursing practice, education, and research

### 5.1 Implications for nursing practice

The Integrated Nurse Self-Leadership and Caring Scale may support nurse managers in assessing professional development needs and strengthening caring cultures within clinical settings. By identifying areas related to self-regulation, motivation, and relational care, the instrument can inform targeted interventions aimed at enhancing professional performance and patient-centered practice. Within the Indonesian healthcare context, structured assessment of integrated self-leadership and caring capacities may contribute to quality improvement initiatives.

### 5.2 Implications for nursing education

In nursing education, the instrument may serve as a formative tool to evaluate the development of self-leadership and caring competencies among undergraduate and professional nursing students. Its use within Indonesian educational programs may support structured reflection and better preparation for the relational and adaptive demands of clinical practice.

### 5.3 Implications for nursing research

For researchers, the scale provides a psychometrically supported outcome measure for examining interventions designed to enhance resilience, self-leadership, and caring capacities. Future studies may extend validation through confirmatory factor analysis and predictive testing linking scores to nurse well-being and patient care outcomes.

## 6 Limitations and future directions

Several limitations should be acknowledged. First, the use of convenience sampling and the inclusion of Indonesian nurses and final-year nursing students may limit the generalisability of the findings to wider nursing populations and to different cultural contexts. Second, although exploratory factor analysis provided preliminary empirical support for the six-domain structure of the Integrated Nurse Self-Leadership and Caring Scale, CFA was not undertaken. The absence of CFA constrains the ability to rigorously validate the proposed measurement model and to examine its stability across independent samples. Third, test–retest reliability was not assessed, leaving the temporal stability of the instrument unexamined. Finally, predictive validity was not evaluated; consequently, the extent to which Integrated Nurse Self-Leadership and Caring Scale scores are associated with key outcomes such as job satisfaction, burnout, and quality of patient care remains to be established.

In addition, although an English translation of the instrument is provided, the English-language version was not tested with English-speaking participants. Therefore, its linguistic clarity and measurement equivalence in English-speaking contexts remain to be established. Future studies should address these limitations by recruiting larger and more diverse samples, conducting CFA to confirm the six-factor structure, and performing test–retest reliability analyses to establish temporal stability. Additionally, predictive validity testing is recommended to examine how Integrated Nurse Self-Leadership and Caring Scale scores are associated with nurse well-being, leadership effectiveness, and patient care outcomes. Such work would not only strengthen the psychometric evidence of the Integrated Nurse Self-Leadership and Caring Scale but also enhance its utility for education, practice, and management in diverse healthcare systems.

## 7 Conclusion

Overall, we found that the Integrated Nurse Self-Leadership and Caring Scale demonstrated promising psychometric properties as an integrated measure of self-leadership and caring in nursing practice in Indonesia. While further validation is required, the instrument represents an important step toward holistic assessment of intrapersonal and interpersonal capacities essential to contemporary nursing.

Holistic assessment of intrapersonal and interpersonal capacities may be important in contemporary nursing because effective patient care depends not only on technical competence but also on emotional regulation, ethical engagement, and relational responsiveness. Instruments that capture these integrated dimensions may contribute to improved patient experiences and strengthened professional well-being among nurses.

## CRediT authorship contribution statement

**Mahmud Ady Yuwanto:** Writing – review & editing, Writing – original draft, Visualization, Validation, Project administration, Methodology, Investigation, Formal analysis, Data curation, Conceptualization. **Iyus Yosep:** Writing – review & editing, Visualization, Validation, Supervision. **Iqbal Pramukti:** Writing – review & editing, Visualization, Validation, Supervision. **Ati Surya Mediawati:** Writing – review & editing, Visualization, Validation, Supervision.

## Declaration of competing interest

The authors declare that they have no known competing financial interests or personal relationships that could have appeared to influence the work reported in this paper.

## Appendix Supplementary materials

Image, application 1
